# Pre-clinical Pharmacokinetic and Metabolomic Analyses of Isorhapontigenin, a Dietary Resveratrol Derivative

**DOI:** 10.3389/fphar.2018.00753

**Published:** 2018-07-11

**Authors:** Yu Dai, Samuel C. M. Yeo, Peter J. Barnes, Louise E. Donnelly, Lai C. Loo, Hai-Shu Lin

**Affiliations:** ^1^Department of Pharmacy, National University of Singapore, Singapore, Singapore; ^2^Airway Disease, National Heart and Lung Institute, Imperial College London, London, United Kingdom; ^3^Shimadzu (Asia Pacific) Pte. Ltd., Singapore, Singapore

**Keywords:** isorhapontigenin, resveratrol, pharmacokinetics, metabolomics, oral bioavailability

## Abstract

**Background:** Isorhapontigenin (*trans*–3,5,4′-trihydroxy–3′–methoxystilbene, ISO), a dietary resveratrol (*trans*–3,5,4′–trihydroxystilbene) derivative, possesses various health-promoting activities. To further evaluate its medicinal potentials, the pharmacokinetic and metabolomic profiles of ISO were examined in Sprague-Dawley rats.

**Methods:** The plasma pharmacokinetics and metabolomics were monitored by liquid chromatography–tandem mass spectrometry (LC–MS/MS) and gas chromatography–tandem mass spectrometry (GC–MS/MS), respectively.

**Results:** Upon intravenous injection (90 μmol/kg), ISO exhibited a fairly rapid clearance (*CL*) and short mean residence time (*MRT*). After a single oral administration (100 μmol/kg), ISO was rapidly absorbed and showed a long residence in the systemic circulation. Dose escalation to 200 μmol/kg resulted in higher dose-normalized maximal plasma concentrations (*C_max_/Dose*), dose-normalized plasma exposures (*AUC/Dose*), and oral bioavailability (*F*). One-week repeated daily dosing of ISO did not alter its major oral pharmacokinetic parameters. Pharmacokinetic comparisons clearly indicated that ISO displayed pharmacokinetic profiles superior to resveratrol as its *C_max_/Dose*, *AUC/Dose*, and *F* were approximately two to three folds greater than resveratrol. Metabolomic investigation revealed that 1-week ISO administration significantly reduced plasma concentrations of arachidonic acid, cholesterol, fructose, allantoin, and cadaverine but increased tryptamine levels, indicating its impact on metabolic pathways related to health-promoting effects.

**Conclusion:** ISO displayed favorable pharmacokinetic profiles and may be a promising nutraceutical in view of its health-promoting properties.

## Introduction

**FIGURE 1:**
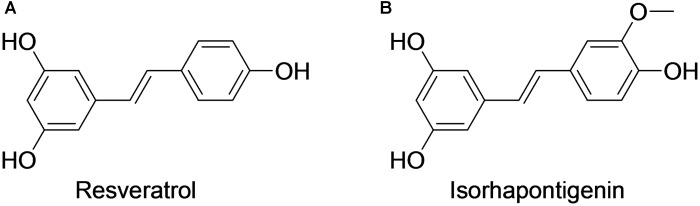
Chemical structures of resveratrol **(A)** and isorhapontigenin **(B)**.

Resveratrol (*trans*-3,4′,5-trihydroxystilbene; **Figure 1**) is a phytoalexin present in a variety of fruits and plants such as grapevines, blueberry, and peanuts (Baur and Sinclair, 2006). Its pleiotropic pharmacological effects have been extensively reported in pre-clinical studies (Baur and Sinclair, 2006). Currently, clinical investigations on the effects of resveratrol in type II diabetes, cardio-protection, cancer chemo-prevention, and Alzheimer’s disease are ongoing. Previous attempts to develop it into a clinically useful drug have been impeded by its weak potency and poor pharmacokinetics.

Isorhapontigenin (*trans*–3,5,4′–trihydroxy–3′–methoxystilbene, ISO; **Figure 1**) is a methoxylated resveratrol derivative present in grapes and some Chinese herbs (Wang et al., 2001; Yao et al., 2003; Fernandez-Marin et al., 2012). Similar to resveratrol, its anti-cancer, anti-oxidation, and cardio-protective activities have been observed in various pre-clinical studies (Wang et al., 2001; Liu and Liu, 2004; Li et al., 2005; Fang et al., 2012, 2013; Zeng et al., 2016; Jiang et al., 2018). In a recent study, the anti-inflammatory effects of ISO were assessed in *in vitro* models for chronic obstructive pulmonary disease (COPD) using resveratrol as a comparator (Yeo et al., 2017). ISO suppressed the activation of several critical inflammatory pathways that are important in COPD (Yeo et al., 2017). Of note, the potency of ISO was much higher than resveratrol and its anti-inflammatory activities were mediated through a mechanistic pathway that is distinct from corticosteroids, which indicates its potential in tackling the corticosteroid-resistant inflammation in COPD (Yeo et al., 2017). As COPD is currently an incurable progressive disease that affects hundreds of millions of people worldwide, it is of great scientific interest to further explore the medicinal potential of ISO.

Pharmacokinetic assessment is an indispensable component in drug development. Although ISO was found to be orally bioavailable after a single administration in two previous studies (Fang et al., 2013; Yeo et al., 2017), more knowledge of its profile following repeated oral administrations is needed, since its promising effects are for chronic medical conditions that require long-term management. Moreover, to optimize its application as therapeutic agent or functional food supplement, the impact of dose on its pharmacokinetics needs to be identified.

Metabolomic studies aim to provide a comprehensive profile of all metabolites present in a biological sample. Such profiles help to explain biological functions or provide detailed biochemical responses of biological systems. Metabolomics has been successfully applied in several fields such as toxicology, medicine, and pharmacology and has recently been extended to nutritional studies (Lindon et al., 2007; Rezzi et al., 2007; Wishart, 2008). Therefore, it is important to assess the metabolomic profile of ISO with the possibility of identifying new health-promoting effects.

In the present study, the pharmacokinetic profiles of ISO were assessed in Sprague-Dawley rats to identify the impacts from dose and repeated dosing on its major pharmacokinetic parameters. A comparison between the major pharmacokinetic parameters of ISO and resveratrol was subsequently executed. Metabolomics was performed using plasma samples collected from rats receiving 1-week daily oral administration of ISO. To our knowledge, this is the first integrated evaluation with pharmacokinetics and metabolomics on ISO. The information obtained from this study will facilitate further development of ISO as a nutraceutical.

## Materials and Methods

### Special Precautions

Due to the light-sensitive nature of stilbene compounds, all laboratory procedures were executed under dimly lit conditions to prevent photo-isomerization of stilbenes (Yeo et al., 2013a,b).

### Chemicals and Reagents

Isorhapontigenin (*trans*-3,5,4′-trihydroxy-3′-methoxystilbene, ISO; **Figure 1**) was purchased from Tokyo Chemical Industry (Tokyo, Japan). Isotopically labeled resveratrol (*trans*-3,5,4′-trihydroxystilbene-^13^C6, mass shift: M + 6; isotopic purity: 99%) and myristic acid (myristic-d_27_ acid, mass shift: M + 27; isotopic purity: 98%) were obtained from Sigma–Aldrich (St. Louis, MO, United States) and used as internal standard (IS) for LC–MS/MS and GC–MS/MS analyses, respectively. The sodium salt of carboxymethylcellulose (CMC), disodium salt of ethylenediaminetetraacetic acid (EDTA), and L-ascorbic acid were supplied by Sigma–Aldrich. 2-Hydroxypropyl-β-cyclodextrin (HP-β-CD; degree of substitution about 0.6) was a kind donation from Wacker (Burghausen, Germany); 2% methoxyamine chloride in pyridine and *N*-methyl-*N*-trifluoroacetamide were obtained from Thermo Fisher Scientific (Waltham, MA, United States); analytical grade DMSO was purchased from MP Biomedical (Santa Ana, CA, United States); HPLC grade acetonitrile and methanol were supplied by Tedia (Fairfield, OH, United States). Ultra-pure water (18.2 MΩ.cm at 25°C) was dispensed from a Millipore Direct-Q^®^ ultra-pure water system (Billerica, MA, United States) and used to prepare mobile phase as well as dosing vehicles. Pooled Sprague-Dawley rat plasma was purchased from Innovative Research (Novi, MI, United States) and used as blank matrix in the preparation of calibration standards and quality control (QC) samples.

### Animals

This *in vivo* study was conducted with strict adherence to the “Guidelines on the Care and Use of Animals for Scientific Purposes” (Singapore). The pharmacokinetic study design and animal handling procedures were reviewed and approved by the Institutional Animal Care and Use Committee of the National University of Singapore (NUS; R15-1273). All animal experiments were carried out in a specific pathogen-free animal facility (temperature: 22 ± 1°C; humidity: 60–70%) in Comparative Medicine, NUS. Sprague-Dawley rats (male, 9–10 weeks old, weight: 300–350 g) were purchased from *InVivos* (Singapore). The rats were housed under a 12 h light–dark cycle with free access to food and water. On the day before the pharmacokinetic study, surgery was performed under isoflurane anesthesia and a catheter (polyethylene tube, i.d. = 0.580 mm, o.d. = 0.965 mm, Becton Dickinson, Sparks, MD, United States) was implanted into the right jugular vein. Intravenous ISO administration and blood collection were carried out via this cannula. To prevent cross-contamination and blood clotting, ∼0.3 mL heparin-saline (10 I.U./mL) was flushed through the cannula after intravenous dosing or each blood collection. This reliable pharmacokinetic model has been routinely used in our laboratory to assess the pharmacokinetic profiles of resveratrol and its derivatives (Yeo et al., 2013a,b, 2017; Choo et al., 2014; Chen et al., 2015, 2016b,c; Dai et al., 2018).

0.3 M HP-β-CD was used to formulate ISO into a solution for intravenous injection while 0.3% CMC (w/v) was applied as a suspending vehicle for oral delivery (Yeo et al., 2017). To enhance the stability of ISO, L-ascorbic acid (final concentration: 0.1 mg/mL) was spiked into both intravenous and oral formulations (Yeo et al., 2017). The concentrations of ISO in intravenous and oral formulations were 5 and 13.3 mg/mL, respectively.

Twenty-two rats were divided into four groups. Group 1 (*n* = 5) received a single bolus intravenous injection of ISO at the dose of 90 μmol/kg (23.2 mg/kg). Serial blood samples were collected before dosing and at 5, 15, 30, 60, 90, 120, 180, 240, 300, 420, 540, and 720 min after intravenous injection. Group 2 (*n* = 5) received a single oral administration of ISO at the dose of 200 μmol/kg (51.6 mg/kg). Serial blood samples were collected before dosing and at 15, 30, 45, 60, 90, 120, 180, 240, 300, 420, 540, and 720 min after oral gavage. Group 3 (*n* = 5) received daily oral pre-treatment of oral dosing vehicle (2 mL/kg) while Group 4 (*n* = 7) received daily oral pre-treatment of ISO at the dose of 100 μmol/kg (25.8 mg/kg) for 1 week. On day 7, ∼8–10 h after the last pre-treatment dose, surgery was performed on rats in Groups 3 and 4 for jugular vein cannulation, and blood samples were also collected from Groups 3 and 4 for metabolomic study. On day 8, the pharmacokinetic study was carried out on these two groups of animals at the dose of 100 μmol/kg (25.8 mg/kg) using the same blood sampling schedule as Group 2.

All blood samples during the pharmacokinetic study were collected in heparinized tubes containing L-ascorbic acid (final concentration = 0.8 mg/mL) to ensure stability of ISO (Yeo et al., 2017). The samples were centrifuged at 5000 *g* for 5 min, and the plasma was harvested and stored at −40°C until LC–MS/MS analysis.

### Quantification of ISO in Rat Plasma by LC–MS/MS Analysis

The LC–MS/MS analysis was carried out using an Agilent 1290 Infinity Liquid Chromatography system (Agilent Technologies, Santa Clara, CA, United States), which is coupled to an ABSciex QTRAP^®^ 5500 mass spectrometer (AB Sciex, Framingham, MA, United States) and equipped with a TurboIon Spray probe (AB Sciex). The LC–MS/MS system was controlled by the Analyst 1.5.2 software (AB Sciex) and chromatographic data analysis was performed using the same software.

Chromatographic separation was achieved at 40°C by a reversed-phase C_18_ column (Agilent Poroshell 120 EC-C18: 75 mm × 3.0 mm, 2.7 μm), which was protected by a guard column (Agilent ZORBAX Eclipse Plus C18 12.5 mm × 4.6 mm, 5 μm) through gradient delivery of a mixture of acetonitrile (A) and water (B) at a flow rate of 0.7 mL/min. The gradient schedule was (a) 0.0–1.0 min A: 20%; (b) 1.0–1.5 min A: 20 → 45%; (c) 1.5–2.5 min A: 45%; (d) 2.5–3.0 min A: 45 → 90%; (e) 3.0–4.0 min A: 90%; (f) 4.0–4.5 min A: 90 → 20%; and (g) 4.5–5.5 min A: 20%.

In the mass spectrometer, nitrogen was used as nebulizing, curtain, and collision gases. The electrospray ionization (ESI) source parameters, including curtain gas, gas 1, gas 2, temperature, and ion spray voltage, were set at 20 psi, 40 psi, 40 psi, 600°C, and −4500 V, respectively. Sequential ramping of the operation potentials identified the optimal compound parameters, comprising of declustering potential (DP), entrance potential (EP), collision energy (CE), and collision exit cell potential (CXP), to be −105.00, −9.00, −26.00, and −11.00 V for ISO and −74.65, −3.90, −26.93, and −12.19 V for IS, respectively. Using the optimized parameters, the MS detector was operated in multiple reaction monitoring (MRM) mode at unit mass resolution. In the mass spectrometer, the most sensitive MRM transitions to be used for quantitation were determined to be *m/z* 257.2 → 241.1 and *m/z* 233.0 → 191.0 for ISO and the IS, respectively. Subsequently, the CE and collision exit potential were optimized to attain the highest sensitivity.

Stock solutions of ISO were prepared by dissolution in DMSO and they were stored at room temperature. The stock solutions were further diluted using blank rat plasma to prepare the calibration standards and QC samples. The stock solution of IS was dissolved in acetonitrile to a concentration of 10 μg/mL. 100 μL IS solution was aliquoted into 1.5-mL polypropylene tubes for storage at −80°C. During sample preparation, this solution was further diluted to the working concentration of 10 ng/mL using acetonitrile containing 0.18 μM of disodium EDTA and stored at 4°C.

The calibration range was 1–1000 ng/mL. A simple protein precipitation procedure was used for sample cleanup prior to LC–MS/MS analysis (Chen et al., 2015, 2016b). Three volumes of 10 ng/mL IS working solution (^13^C isotopically labeled resveratrol in acetonitrile–0.18 μM disodium EDTA) were added to one volume of plasma sample spiked with of L-ascorbic acid at a final concentration of 0.8 mg/mL. After vigorous vortex, centrifugation of samples was performed at 15,000 *g* for 10 min at 4°C. Subsequently, the clear supernatants were transferred into HPLC vials. During each LC–MS/MS analysis, 1 μL of supernatant was injected into the system.

This LC–MS/MS has been successfully applied in our recent study (Yeo et al., 2017).

### Pharmacokinetic Calculations

All pharmacokinetic analyses were performed with WinNonlin standard version 1.0 (Scientific Consulting, Apex, NC, United States). As “double-peak phenomenon” was observed in plasma ISO–time curve, the apparent volume of distribution (*V*) was estimated with the one-compartment first-order open model (C = C_0_ ⋅ e^−k⋅t^) using the data obtained from the first hour (Chen et al., 2016c; Yeo et al., 2017). All other pharmacokinetic parameters were calculated through non-compartmental analyses. The clearance (*CL*) and mean residence time (*MRT*) were also calculated through non-compartmental analyses without an extrapolation to infinity (Chen et al., 2016a,c). The absolute oral bioavailability (*F*) of ISO was calculated using the following equation:

$$F(\%)=\frac{{\text{AUC}}_{0\to \text{t}}(\text{oral})}{\text{Oral}\;\text{Dose}}÷\frac{\text{AU}{\text{C}}_{0\to \text{t}}(\text{mean}\;\text{value}\;\text{of}\;\text{Group}\;1)}{23.2\;\text{mg}/\text{kg}}\times 100\%$$

### Pharmacokinetic Comparisons

All pharmacokinetic comparison was carried out using GraphPad Prism 7.03 (GraphPad Software, Inc., La Jolla, CA, United States). The normality of data distribution was first assessed with Kolmogorov–Smirnov test. If the pharmacokinetic parameters follow Gaussian (normal) distribution, two-tailed unpaired *t*-test is chosen; otherwise, Mann–Whitney test will be implemented. A *p*-value less than 0.05 indicates statistically significant difference.

A meaningful pharmacokinetic comparison between ISO and resveratrol was also executed using the resveratrol pharmacokinetic data extracted from a recent study, where the pharmacokinetic profiles of resveratrol were examined using the same animal model, molar doses, similar formulations, and analytical methods (Chen et al., 2016c; Data Reuse License Number: 4330720025182).

### Metabolomic Profiling of ISO in Rat Plasma by GC–MS/MS Analysis

The plasma samples collected from rats receiving 1-week daily oral dosing of ISO at 100 μmol/kg (25.8 mg/kg) were subjected to metabolomic study using the rats receiving vehicle as control.

Plasma samples (30 μL aliquots in Eppendorf tubes) were thawed to room temperature (25°C). Myristic-d_27_ acid (20 μL of 200 μg/mL solution in methanol, IS) and 200 μL of methanol were added to each sample. Samples were vortexed for 5 min at room temperature and centrifuged for 10 min at 15,000 *g* (4°C). The top 200 μL of each supernatant was transferred to a glass centrifuge tube and dried under nitrogen gas. The dried samples were re-suspended in 100 μL of toluene, vortexed vigorously for 10 s, and dried again under nitrogen. A two-step derivatization method was used for chemical derivatization of the metabolites. Samples were first incubated with 50 μL of 2% methoxy-amine chloride in pyridine for 1.5 h at 60°C. Next, 50 μL of *N*-methyl-*N*-trifluoroacetamide was added and the samples were incubated again for 1 h at 60°C. A total of 80 μL of derivatized samples were transferred to glass vials for GC–MS/MS analysis (Chen et al., 2016a). Pooled rat plasma from different plasma samples was used as QC and distilled water as blank samples. Two pooled plasma samples were injected before the first study sample and three pooled plasma samples were injected after every six study samples which serve as QC. The blank samples were injected at the beginning, at the middle, and at the end of the sample batch (Phua et al., 2015; Chen et al., 2016a).

GC–MS/MS analysis was performed using a Shimadzu TQ8040 gas chromatography-triple quadrupole mass spectrometer (Shimadzu Corporation, Japan). The GC injector port was set at 250°C and injection volume was 1 μL with a spilt ratio of 1:10. Chromatographic separation was achieved by a capillary column (BPX-5, 30 m × 0.25 mm × 0.25 μm, SGE Analytical Science, Australia) through constant helium carrier gas flow at 1.14 mL/min. The oven temperature was first held at 60°C for 2 min, followed by increasing temperature ramp of 15°C/min to 330°C, and then held at 330°C for 3 min. The interface and ion source were set at 280 and 200°C, respectively. The mass spectrometer was operated in the MRM mode.

### Metabolite Identification and Data Processing

The Shimadzu Smart Metabolites Database (Shimadzu Corporation, Japan) which contains a panel of 475 different endogenous metabolites was applied to identify the metabolites in rat plasma samples in this widely targeted metabolomics analysis. This database contains the retention indices and two MRM transitions of each of the 475 endogenous metabolites which facilitate simple, fast, and reliable metabolite identification and semi-quantification. This strategy has been successfully applied to many studies (Hashimoto et al., 2017; Nishiumi et al., 2017; Tomonaga et al., 2018). Metabolites existing in the real samples but not in blank samples or metabolites in real samples with peak areas at least fivefold as high as that in blank samples were selected for further data analysis. In addition, metabolites with ion transition ratio variation > 30% and/or with peak area variation in QC samples > 30% were removed from the analysis list as they were considered unstable (Chen et al., 2016a).

The raw data were normalized with the peak area of IS and the final data were imported into the SimCA 13 software (Umetrics AB, Umea, Sweden). Principal component analysis (PCA) was performed via auto-scaling to visualize general clustering, trends, and outliers among all samples on the score plot. Next, the data were subjected to partial least squares discriminant analysis (PLS-DA). The PLS-DA model was validated by cross-validation techniques and permutation tests (100 repetitions). Metabolites with variable importance on projection (VIP) value higher than 1 was viewed as critical and subjected to two-tailed unpaired *t*-test with a *p*-value of 0.05 indicating statistical significance (Phua et al., 2015). Furthermore, false discovery rates (FDRs) were calculated by Benjamini–Hochberg procedure. Metabolites with FDR < 0.1 and fold change > 1.15 or < 0.85 were accepted as significant. Significant metabolites were submitted to MetaboAnalyst for pathway analysis.

## Results and Discussion

### Validation of LC–MS/MS

**FIGURE 2:**
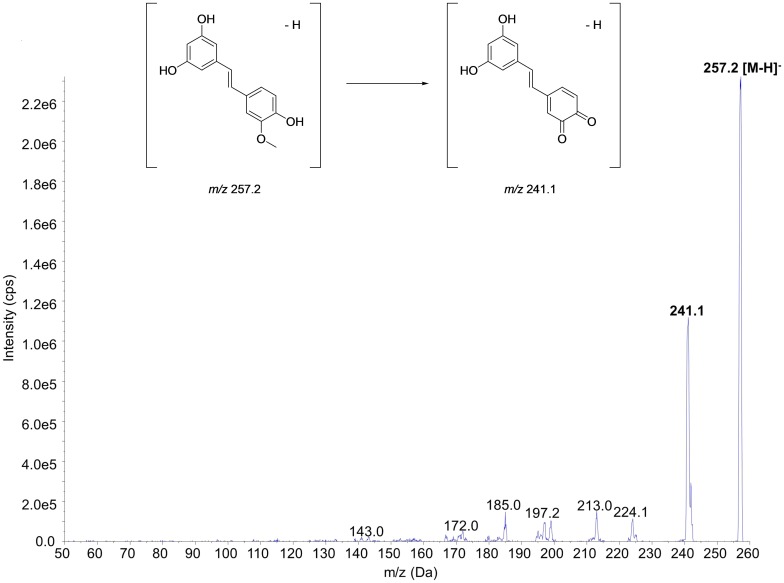
MS/MS spectra of isorhapontigenin and proposed fragmentation pattern of *m/z* 257.2 → 241.1 transition.

The mass spectra of ISO and the proposed fragmentation pattern of *m/z* 257.2 → 241.1 transition are shown in **Figure 2**. Of note, this MRM transition has been applied to quantify ISO although the details were not disclosed (Fang et al., 2013; Yeo et al., 2017). Similar data for the IS can be found in some previous studies (Yeo et al., 2013b; Chen et al., 2015).

**FIGURE 3:**
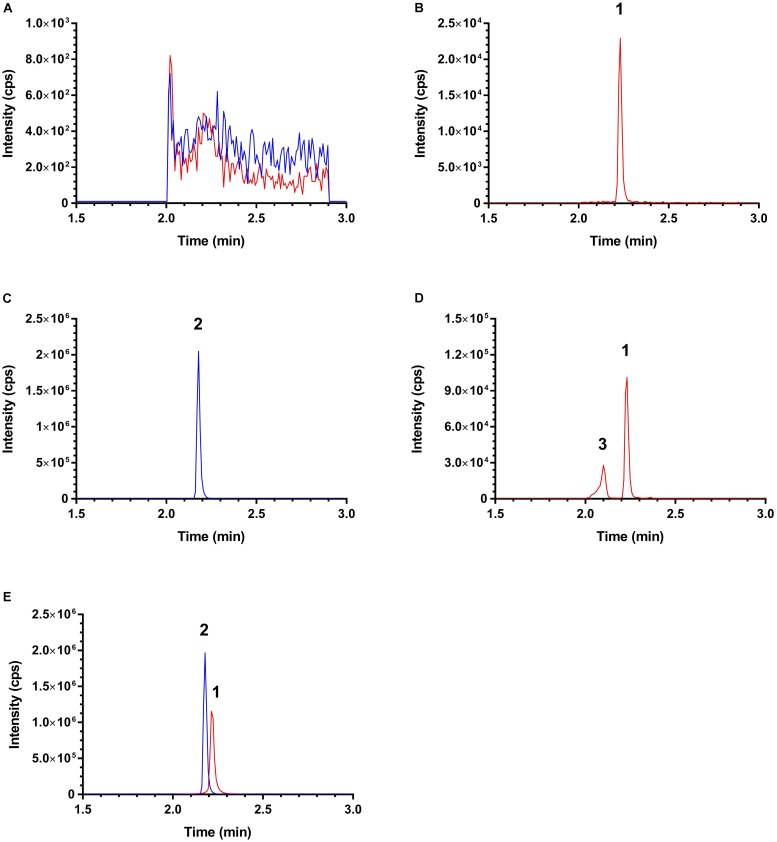
Typical MRM chromatograms of **(A)** a pre-dosing plasma sample; **(B)** a blank plasma sample spiked with 3 ng/mL isorhapontigenin; **(C)** a blank plasma sample spiked with 30 ng/mL internal standard; **(D)** a plasma sample collected 90 min after intravenous injection; and **(E)** a plasma sample collected 180 min after oral dosing. Red: MRM precursor-to-product ion transition of *m/z* 257.2 → 241.1 and blue: MRM precursor-to-product ion transition of *m/z* 241.0 → 181.0. Peak 1, isorhapontigenin; peak 2, internal standard; and peaks 3, unidentified metabolite.

The selectivity of this LC–MS/MS was demonstrated as no notable interference was observed at the retention times of ISO and IS at MRM channels *m/z* 257.2 → 241.1 (for ISO) and *m/z* 233.0 → 191.0 (for IS) in the chromatograms from the blank rat plasma samples/pre-dosing plasma samples and post-dosing samples collected from pharmacokinetic study. Typical MRM chromatograms of a pre-dosing plasma sample, a blank plasma sample spiked with 3 ng/mL ISO, a blank plasma sample spiked with 30 ng/mL IS, and plasma samples collected after respective ISO oral and intravenous dosing are shown in **Figure 3**. An unidentified ISO metabolite eluting at ∼2.1 min was observed (peak 3, **Figure 3D**). Interestingly, this metabolite also shares the *m/z* 257.2 → 241.1 transition. As this metabolite is obviously more polar than ISO, it might be a glucuronide or sulfate generated by phase II metabolism. Conjugated metabolites such as glucuronides and sulfates are generally quite unstable and could be converted and ionized in a similar way as ISO during ESI. However, such postulation needs to be confirmed in future with authentic standards.

This LC–MS/MS method also displayed excellent accuracy and precision. The stability of ISO was also tested and ISO was found to be quite stable under the experimental conditions. Matrix effect was found to be insignificant as the variation in terms of relative standard deviation (coefficient of variation) was less than 15%. The dilution integrity was confirmed using a dilution factor of 20. In the presence of L-ascorbic acid (0.8 mg/mL), ISO appeared to stable in plasma; similarly, addition of EDTA in IS acetonitrile solution (0.18 μM) enhanced the post-preparative stability. The details in accuracy, precision, stability, and matrix effect can be found in Supplementary Tables S1–S3.

As seen, a rapid and sensitive LC–MS/MS method has been developed and rigorously validated for the quantitation of ISO in rat plasma.

### The Impact of Dose and Repeated Dosing on the Pharmacokinetics of ISO

**FIGURE 4:**
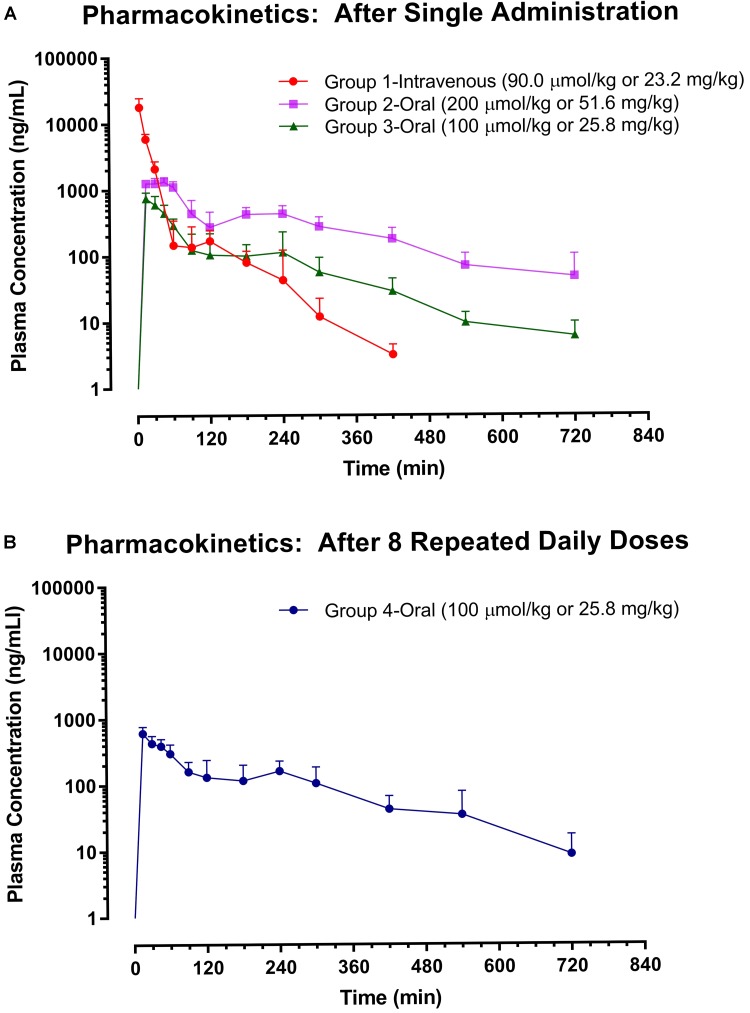
Pharmacokinetics of isorhapontigenin. Plasma isorhapontigenin levels were measured with LC–MS/MS. Symbols represent mean concentrations and error bars represent SD. **(A)** Groups 1 – 3: *n* = 5; except Group 2 at 720 min where *n* = 4. **(B)** Group 4: *n* = 7.

Although the pharmacokinetics of ISO in Sprague-Dawley rats has been performed previously after respective single intravenous (30 μmol/kg) and oral (600 μmol/kg) administration (Yeo et al., 2017), to identify the impacts of dose and repeated dosing on the major pharmacokinetic parameters of ISO and to facilitate a fair pharmacokinetic comparison between ISO and resveratrol, the pharmacokinetic examination of ISO was extended in the present study with four groups of animals, which received higher single intravenous dose (Group 1: 90 μmol/kg), lower single oral dose (Group 2: 200 μmol/kg; Group 3: 100 μmol/kg), and eight repeated daily oral doses (Group 4: 100 μmol/kg). The plasma levels of ISO were measured by LC–MS/MS method and the plasma ISO profiles are displayed in **Figure 4** while the major pharmacokinetic parameters are listed in **Table 1**.

**Table 1:** Pharmacokinetic profiles of isorhapontigenin^∗^.

Parameters	Intravenous		Oral administration	
Grouping	1 (*n* = 5)	2 (*n* = 5)	3 (*n* = 5)^a^	4 (*n* = 7)^b^
Dose (μmol/kg)	90	200	100	100
Formulation	HP–β–CD	Suspension	Suspension	Suspension
*V* (mL/kg)	893 ± 103	–	–	–
*CL* (mL/min/kg)	76.8 ± 23.7	–	–	–
*t_max_* (min)	–	15–45	15	15 or 30
*C_max_ /Dose* (g/L)	–	27.8 ± 3.2	29.2 ± 2.8	23.9 ± 6.2
*AUC_0→last_ /Dose* (kg⋅min/L)	14.2 ± 5.2	4.28 ± 0.94^#^	2.54 ± 0.69	2.97 ± 0.80
*F* (%)	–	30.1 ± 6.6^#^	17.9 ± 4.9	20.8 ± 5.6
*MRT* (min)	18.8 ± 3.2	184 ± 23^#^	136 ± 29	182 ± 42

*^∗^Data are expressed as mean ± SD. ^a^Rats in Group 3 received 1-week daily oral pre-treatment of vehicle. ^b^Rats in Group 4 received 1-week daily oral pre-treatment of isorhapontigenin at the dose of 100 μmol/kg. ^#^p < 0.05 between Group 3 and this group.*

After single intravenous injection at 90 μmol/kg (Group 1), a rapid distribution followed by a prolonged elimination phase was observed in plasma ISO–time curve (**Figure 4**). ISO possessed a moderate apparent volume of distribution (*V* = 893 ± 103 mL/kg), quite rapid clearance (*CL* = 76.8 ± 23.7 mL/min/kg), and short mean residence time (*MRT* = 18.8 ± 3.2 min). The rapid elimination of ISO concurred with the structural-pharmacokinetic relationship identified in previous studies, i.e., stilbene compounds with meta-hydroxyl group(s), e.g., desoxyrhapontigenin (*trans*-3,5-dihydroxy-4′-methoxystilbene), pinostilbene (*trans*-3,4′-dihydroxy-5-methoxystilbene), pinosylvin (*trans*-3,5-dihydroxystilbene), oxyresveratrol (*trans*-3,5,2′,4′-tetrahydroxystilbene), and resveratrol, are subjected to rapid clearance (Yeo et al., 2013b; Chen et al., 2016b,c; Dai et al., 2018).

A secondary peak was observed at ∼2 h after intravenous administration. Such a phenomenon might be due to entero-hepatic circulation in which has similarly been observed with resveratrol (Marier et al., 2002; Chen et al., 2016c). When compared to the pharmacokinetic data obtained at a lower intravenous dose (30 μmol/kg; Yeo et al., 2017), the dose escalation to 90 μmol/kg slightly increased the *MRT* (two-tailed unpaired *t*-test: *p* = 0.0318) but not the *V* and *CL* (two-tailed unpaired *t*-test: *p* > 0.05).

After single oral administration at 100 μmol/kg (Group 3), ISO displayed very rapid absorption as the maximal plasma concentration (*C_max_*) was achieved at 15 min, the first post-dosing sampling time point (**Figure 4**). As rapid oral absorption was observed even with suspension formulation, aqueous solubility was unlikely to be a barrier to its oral absorption. Of note, the aqueous solubility was commonly shown to be a barrier to the oral absorption of polymethoxylated stilbenes (Lin and Ho, 2011; Yeo et al., 2013a). Although the *F* of ISO was not high (17.9 ± 4.9%), it remained measurable in the systemic circulation until at least 12 h after administration. Dose escalation to 200 μmol/kg resulted in a longer time to *C_max_* (*t_max_*) (15–45 versus 15 min, Mann–Whitney test: *p* = 0.0476), *MRT* (184 ± 23 *versus* 136 ± 29 min, two-tailed unpaired *t*-test: *p* = 0.0221), and an increased *F* (30.1 ± 6.6 *versus* 17.9 ± 4.9 %, two-tailed unpaired *t*-test: *p* = 0.0103). The increases in *MRT* and *F* may be due to saturation in the elimination of ISO at higher doses. However, 1-week oral pre-treatment of ISO at 100 μmol/kg did not alter its major pharmacokinetic parameters including *C_max_*, *MRT*, *AUC_0→last_*, and *F* (two-tailed unpaired *t*-test: *p* > 0.05). Clearly, there was no auto-induction of ISO metabolism which renders dose adjustment unnecessary during its long-term application.

### Pharmacokinetic Comparison Between ISO and Resveratrol

**FIGURE 5:**
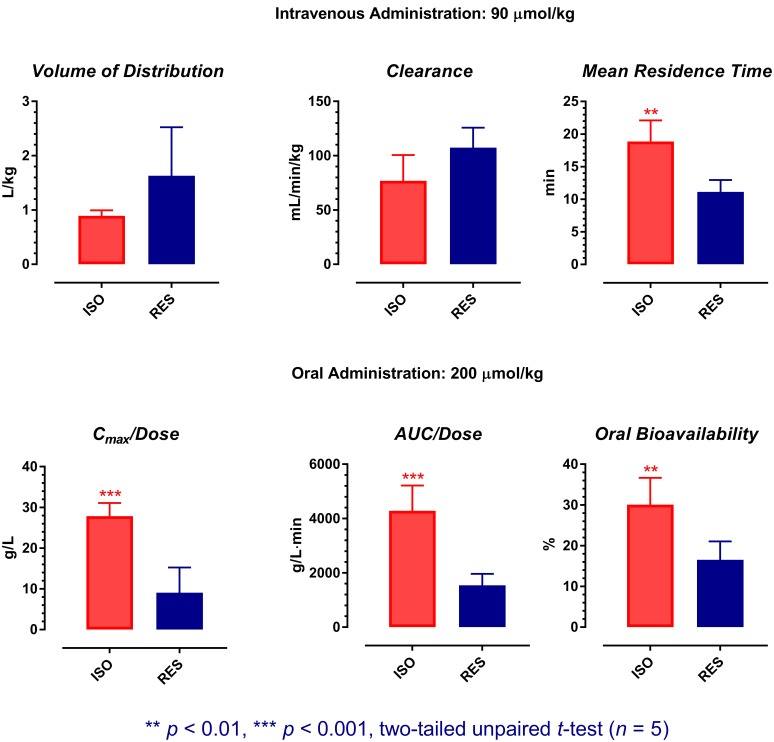
Pharmacokinetic comparison between isorhapontigenin and resveratrol. The pharmacokinetic data of resveratrol was extracted from a previous report with permission (Data Reuse License Number: 4282330274888). ISO, isorhapontigenin; RES, resveratrol. Symbols represent mean values, while error bars represent SD.

A preliminary pharmacokinetic comparison between ISO and resveratrol has been performed recently (Yeo et al., 2017). However, as ISO possessed dose-dependent pharmacokinetics, pharmacokinetic comparison using data obtained at different doses may not be meaningful. Therefore, the pharmacokinetic profiles of ISO were re-examined in this study at the molar doses previously applied for resveratrol (intravenous: 90 μmol/kg; oral: 200 μmol/kg). As the same animal model, molar doses, similar formulations, and analytical methods were applied in both studies, an accurate pharmacokinetic comparison between ISO and resveratrol is assured. The results of statistical analyses are shown in **Figure 5**.

After single intravenous administration, although the *V* and *CL* of ISO were comparable to resveratrol (two-tailed unpaired *t*-test: *p* > 0.05), its average *MRT* was ∼70% longer than resveratrol (two-tailed unpaired *t*-test: *p* = 0.0016). For the management of chronic conditions such as COPD and cardiovascular diseases, oral administration is the most convenient route for drug delivery. Therefore, the oral pharmacokinetic profiles of ISO and resveratrol are more crucial for evaluation. After single oral dosing, although both ISO and resveratrol were absorbed rapidly, ISO displayed an oral pharmacokinetic profile that is much more favorable than resveratrol as its average dose normalized *C_max_* (*C_max_/Dose*), dose normalized *AUC_0→t_* (*AUC_0→t_/Dose*), and *F* were about two to three folds greater than that of resveratrol (two-tailed unpaired *t*-test: *p* at least < 0.01). This suggests that from the pharmacokinetic perspective, ISO is a better candidate for drug/nutraceutical development.

### Metabolomic Study

Despite its relative weak pharmacological potencies and inferior pharmacokinetics, resveratrol is becoming a popular functional food supplement or nutraceutical. Therefore, it is of interest to identify dietary resveratrol derivatives, which may possess superior potencies and more favorable oral bioavailability. ISO appears to be an excellent candidate for functional food supplement/nutraceutical as its superior anti-inflammatory activity and oral bioavailability have been confirmed. Therefore, in the present study, the biological activities of ISO were subsequently probed using a metabolomic approach.

### Plasma Metabolomics Data Processing

**FIGURE 6:**
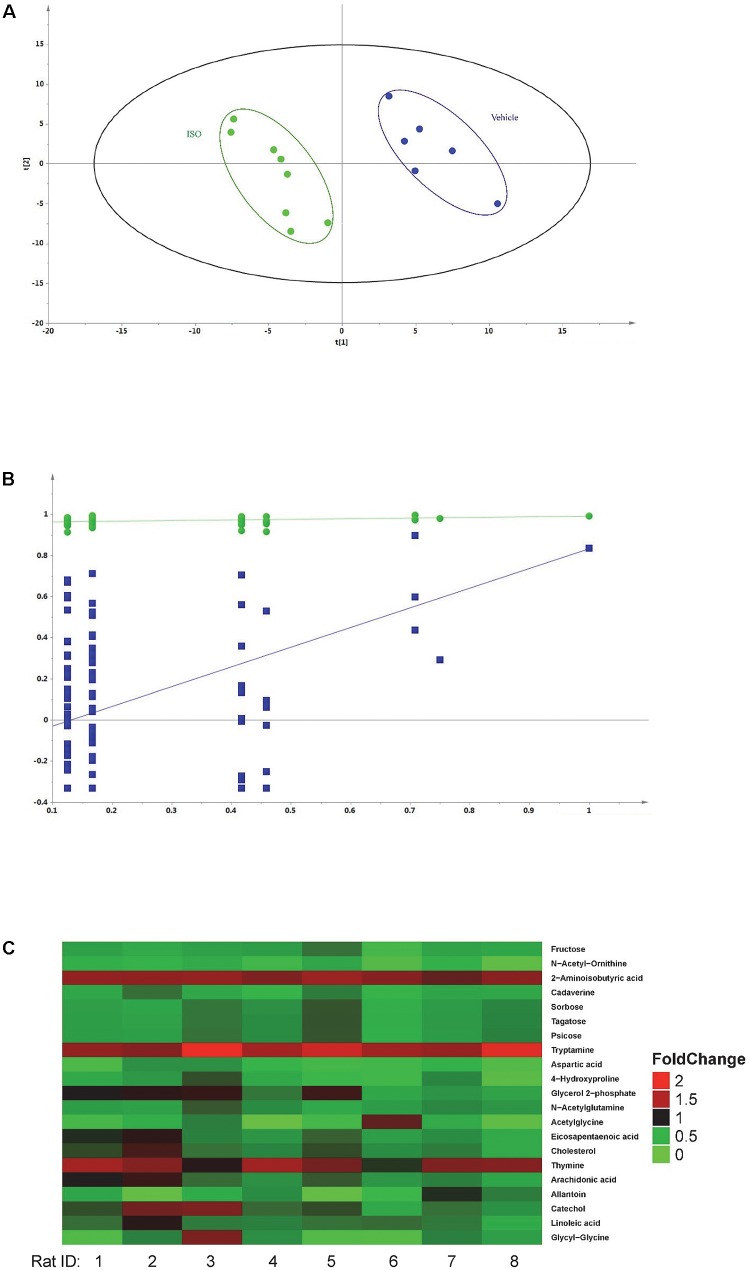
Metabolomics of isorhapontigenin. **(A)** Results of PLS-DA. Green •: rats received 1-week ISO intervention and blue •: rats received vehicle. **(B)** Results of permutation test. Green •: *R*^2^ (goodness-of-fit parameter) and blue ■: Q^2^ (goodness-of-prediction parameter). **(C)**: Fold change heatmap of plasma metabolites altered by 1-week ISO intervention.

Fourteen samples were analyzed by GC-MS/MS (six samples were from vehicle control group and eight samples were from ISO treated group). The peak areas of individual metabolites were normalized by the peak area of the IS. Differences between ISO and vehicle group were analyzed by the PLS-DA model. As shown in **Figure 6A**, the PLS-DA model differentiated the rats that had received 1-week ISO treatment from the rats that received vehicle (two component, *R*^2^*X* = 0.362, *R*^2^*Y* = 0.956, *Q*^2^ = 0.701, *p* = 0.02). The two groups of rats were well separated in the PLS-DA scores plot, with ISO-treated group located on the left side while the vehicle group on the right side. Model validation was performed by permutation test and the *Q*^2^ values of all 100 different permutations were lower than those of actual sample classifications (**Figure 6B**) while the *y* intercept of regression line of *Q*^2^ was below zero, which indicates the validity of the PLS-DA model (Phua et al., 2015).

### Identification of Metabolic Alternation

Based on the globally accepted standard, alteration in metabolites was considered as critical when its corresponding VIP > 1, FDR < 0.1, and fold change > 1.15 or < 0.85. Twenty-one metabolites were identified as critical metabolites related to ISO intervention, their statistic data, fold change, retention time, and MS transition 1 and MS transition 2 are listed in **Table 2**. The fold change heat map suggested the administration of ISO caused a broad decrease in critical metabolite content (**Figure 6C**). Metabolic pathway analysis showed sugar metabolism (starch and sucrose metabolism and amino sugar and nucleotide sugar metabolism), fatty acid biosynthesis (biosynthesis of unsaturated fatty acids), amino acid metabolism (beta-alanine, arginine, proline, alanine, aspartate, glutamate and tryptophan metabolism), primary bile acid biosynthesis, linoleic acid metabolism, arachidonic acid metabolism, and pyrimidine metabolism were influenced after ISO intervention.

**Table 2:** Change in plasma metabolites after 1-week isorhapontigenin intervention.

Name	*p*-value	FDR	Fold change	Retention time (min)	MS transition1	MS transition 2
Fructose	<0.001	0.001	0.608	13.418	307.10 > 217.20	307.10 > 73.00
N-Acetyl-ornithine	<0.001	0.001	0.474	14.160	174.10 > 73.00	200.10 > 73.00
2-Aminoisobutyric acid	<0.001	0.001	1.351	7.239	130.10 > 73.00	204.10 > 147.10
Cadaverine	<0.001	0.002	0.54	11.627	174.10 > 73.00	174.10 > 86.00
Sorbose	<0.001	0.002	0.698	13.341	307.10 > 217.10	307.10 > 73.00
Tagatose	<0.001	0.003	0.705	13.193	307.10 > 217.10	307.10 > 103.10
Psicose	<0.001	0.003	0.709	13.242	307.10 > 217.10	307.10 > 73.00
Tryptamine	0.002	0.013	1.469	14.294	102.10 > 73.00	203.10 > 73.00
Aspartic acid	0.002	0.017	0.475	10.789	232.10 > 73.00	218.10 > 73.00
4-Hydroxyproline	0.004	0.025	0.564	10.855	230.10 > 73.00	230.10 > 140.10
Glycerol 2-phosphate	0.004	0.025	0.73	12.330	299.10 > 73.00	315.10 > 73.00
N-Acetylglutamine	0.007	0.032	0.804	13.797	274.20 > 73.00	243.20 > 73.00
Acetylglycine	0.009	0.038	0.508	8.834	145.20 > 130.10	174.10 > 75.00
Eicosapentaenoic acid	0.01	0.038	0.762	16.764	117.10 > 75.00	117.10 > 115.10
Cholesterol	0.014	0.049	0.789	20.724	458.40 > 119.10	458.40 > 201.20
Thymine	0.015	0.052	1.198	9.831	255.10 > 147.10	270.20 > 255.10
Arachidonic acid	0.02	0.066	0.789	16.719	117.10 > 75.00	93.10 > 77.00
Allantoin	0.026	0.08	0.545	13.586	331.20 > 73.00	431.20 > 201.10
Catechol	0.027	0.079	1.411	9.085	254.10 > 73.00	239.10 > 73.00
Linoleic acid	0.029	0.08	0.82	15.787	337.30 > 75.00	337.30 > 131.10
Glycyl-glycine	0.035	0.092	0.648	13.057	248.10 > 147.10	405.20 > 147.10

Although ISO intervention led to a drop in the concentrations of a variety of sugars including fructose, sorbose, tagatose, and psicose, it did not change glucose levels. This finding was consistent with a recent study, where a single intravascular dose of ISO neither decreased blood glucose nor improved insulin resistance (Oritani et al., 2016). Although ISO may not be useful in diabetes, as it downregulated fructose and sorbose, it might have some effect in other metabolic disorders such as fatty liver disease. Of note, no food restriction was applied before blood sampling in this study. It is more appropriate to collect the blood samples after over-night fasting in future study.

Interestingly, 1-week daily oral dosing of ISO also significantly decreased plasma cholesterol level, suggesting its medicinal potential in hypercholesterolemia and/or other cardiovascular diseases. This finding was also in agreement with another recent study, where ISO displayed *in vivo* cardio-protective effects in rat isoproterenol-induced myocardial infarction model (Abbas, 2016). Moreover, as ISO intervention also downregulated glycerol 2-phosphate, eicosapentaenoic acid, linoleic acid, and arachidonic acid, it may also have significant impact in hyperlipidemia.

Cadaverine is a diamine produced by putrefaction in animal tissue (Kim et al., 2018). Elevation of cadaverine has been proposed to be a biomarker for cancers and some inflammatory diseases such as systemic lupus erythematosus and periodontitis (Liu et al., 2017; Kim et al., 2018). Moreover, a pro-inflammatory role of cadaverine was observed in a recent *in vivo* study (Fan et al., 2012). Similarly, arachidonic acid is a polyunsaturated fatty acid which plays an important role in mediation of a variety of inflammation responses and initiation of oxidative stress via serving as the precursor for the synthesis of eicosanoids and prostanoids (Ng et al., 2017). In this study, 1-week ISO intervention was found to downregulate plasma levels of both cadaverine and arachidonic acid. Such findings correlated well with the anti-inflammatory effects of ISO, which have been observed in previous studies (Abbas, 2016; Yeo et al., 2017).

Allantoin, a non-enzymatic oxidative product of uric acid in humans, can be used as a biomarker for oxidative stress (Zitnanova et al., 2004; Gruber et al., 2009). The decrease in plasma allantoin levels suggests that ISO was able to ameliorate oxidative stress *in vivo*. Similarly, daily oral administration of an extract of *Polygonum cuspidatum* (standardized at 20% resveratrol; 100 mg/kg) for 7 weeks was also found to down-regulate urinary level of allantoin (Peron et al., 2017). The metabolic data are concordant with the reported anti-oxidant activities of ISO in previous studies where such activities contributed to its efficacy in various disease models (Liu and Liu, 2004; Li et al., 2005; Abbas, 2016) and appear to be a common property of other polyhydroxystilbenes such as resveratrol (Baur and Sinclair, 2006), oxyresveratrol (Choi et al., 2016), and piceatannol (Seyed et al., 2016).

4-Hydroxyproline is one of the major components of collagen (Shoulders and Raines, 2009). Since elevated urinary excretion of 4-hydroxyproline is commonly associated with accelerated bone resorption, 4-hyroxyproline is well accepted as a biomarker for bone turnover and osteoporosis (Lofman et al., 2005). As ISO intervention decreased plasma 4-hydroxyproline, it might offer beneficial effects in bone health, e.g., management of osteoporosis and rheumatic diseases.

Tryptamine, a metabolite of tryptophan, is a neurotransmitter or a neuromodulator. It plays an important physiological role in the neuronal system but is also implicated in various neuropsychiatric disorders (Mousseau, 1993; Shimazu and Miklya, 2004). One-week ISO intervention significantly elevated plasma level of tryptamine, suggesting that ISO may possess some neuropsychopharmacological activities. Of note, the anti-depressant activities of RES have been extensively reported (de Oliveira et al., 2017). Clearly, it is of interest to assess the therapeutic potential of ISO in depression.

One-week daily oral dosing of ISO decreased plasma levels of some di-aminoacid and *N*-acetyl amino acids such as glycyl-glycine, *N*-acetyl-glycine, *N*-acetyl-glutamine, and *N*-acetyl-orthinine. Specifically, *N*-acetyl-glycine and *N*-acetyl-orthinine have been found to be associated with weight gain and/or obesity (Wang et al., 2011; Zhao et al., 2016). In this study, the healthy Sprague-Dawley rats were fed with normal diet. Such a model may not be appropriate for examining the anti-obesity effect of ISO and, the body weights of the rats in both the vehicle and ISO treatment groups remained similar before and after ISO intervention (two-tailed unpaired *t*-test, *p* = 0.74 and 0.89, respectively). To examine the impact of ISO, resveratrol, or other resveratrol analogs on obesity and other metabolic disorders, a special diet, e.g., fat-rich diet should be tested for a longer period. Besides obesity, elevation of glycyl-glycine, *N*-acetyl-glycine, *N*-acetyl-glutamine, and *N*-acetyl-orthinine in body tissue/fluids has been observed in a variety of pathological conditions (Racine et al., 2004; Carrola et al., 2011; Manna et al., 2011; Ouyang, 2012; Kim et al., 2013; Li et al., 2016; Schnackenberg et al., 2016). Therefore, downregulation of these biomarkers by IOS may provide health-promoting effects.

In summary, the anti-oxidative, anti-inflammatory, and cholesterol-lowering effects of ISO were well supported by the metabolomic study. This study also suggested that ISO may have medicinal potentials in obesity, bone health, and neuropsychiatric disorder.

## Conclusion

In this study, the pharmacokinetic profiles of ISO were assessed in Sprague-Dawley rats. The plasma metabolomics were also profiled using plasma samples collected after 1-week daily oral administration of ISO. Favorable pharmacokinetic characteristics, including rapid absorption, long systemic residence, and unaltered exposure after repeated oral dosing, were identified and ISO was found to display pharmacokinetic profiles superior to resveratrol. The multiple health-promoting roles of ISO were well supported by the metabolomic study. As ISO possessed beneficial biological activities and favorable pharmacokinetic profiles, it appears to be a promising nutraceutical for various chronic medical conditions.

## Author Contributions

SY developed and validated the LC-MS/MS method. H-SL and YD carried out all *in vivo* work. YD analyzed the plasma samples with LC-MS/MS and performed the metabolomic data analysis. H-SL performed all pharmacokinetic calculations. YD, SY, and LL analyzed the metabolomic samples with GC-MS/MS. YD, SY, PB, LD, and H-SL wrote the manuscript. All authors read and approved the final version of the manuscript.

## Conflict of Interest Statement

The authors declare that the research was conducted in the absence of any commercial or financial relationships that could be construed as a potential conflict of interest.
