# Supplementary material for: Pre-clinical Pharmacokinetic and Metabolomic Analyses of Isorhapontigenin, a Dietary Resveratrol Derivative
Source: Front Pharmacol. 2018 Jul 11;9:753. doi: 10.3389/fphar.2018.00753 (PMC6050476; doi:10.3389/fphar.2018.00753)
Supplement: Supplementary file 1 [file Table_1.DOC]

| S-Table 1. Intra-day and inter-day accuracy ^a^ | | | |
| --- | --- | --- | --- |
| **Parameters** | **Concentrations of QC samples (ng/ml)** | | |
|  | **2** | **400** | **800** |
| Intra-day (*n* = 5) |  |  |  |
| Analytical recovery (%) | 102.9 ± 7.3 | 105.9 ± 7.2 | 101.9 ± 4.9 |
| CV (%) | 7.2 | 6.8 | 4.8 |
| Inter-day (*n* = 3) |  |  |  |
| Analytical recovery (%) | 93.1 ± 9.4 | 92.9 ± 7.7 | 91.2 ± 3.7 |
| CV (%) | 10.1 | 8.2 | 4.1 |
| ^a^ Data is presented as mean ± SD | | | |
