# Supplementary material for: Pre-clinical Pharmacokinetic and Metabolomic Analyses of Isorhapontigenin, a Dietary Resveratrol Derivative
Source: Front Pharmacol. 2018 Jul 11;9:753. doi: 10.3389/fphar.2018.00753 (PMC6050476; doi:10.3389/fphar.2018.00753)
Supplement: Supplementary file 2 [file Table_2.DOC]

S-Table 2 Matrix factor of isorhapontigenin in rat plasma ^a^

| **Matrix factor** | **Concentrations of QC samples (ng/ml)** | |
| --- | --- | --- |
|  | **2** | **800** |
| Mean (%) | 1.203 ± 0.043 | 1.218 ± 0.036 |
| CV (%) | 3.6 | 3.0 |
| ^a^ Data presented as mean ± SD (*n* = 5) | | |
