# Supplementary material for: Pre-clinical Pharmacokinetic and Metabolomic Analyses of Isorhapontigenin, a Dietary Resveratrol Derivative
Source: Front Pharmacol. 2018 Jul 11;9:753. doi: 10.3389/fphar.2018.00753 (PMC6050476; doi:10.3389/fphar.2018.00753)
Supplement: Supplementary file 3 [file Table_3.DOC]

S-Table 3 Stability of isorhapontigenin under different conditions ^a^

| **Storage conditions**  **(% remaining)** | **Concentrations of QC samples (ng/ml)** | | |
| --- | --- | --- | --- |
|  | **2** | **400** | **800** |
| Short term (on ice, 3 h) | 104.0 ± 6.0 | 102.0 ± 5.4 | 98.7 ± 6.4 |
| Long term (-80 °C, 14 days) | 87.9 ± 2.5 | 101.0 ± 3.0 | 97.0 ± 6.5 |
| Post preparative (4 °C, 6 h) | 108.0 ± 6.1 | 108.0 ± 1.0 | 108.0 ± 4.1 |
| Freeze thaw (3 cycles) | 98.4 ± 7.6 | 94.0 ± 2.4 | 92.3 ± 1.2 |
| Stock solution stability (24 °C, 10 days) |  | 96.7 ± 3.0 |  |
| ^a^ Data presented as mean ± SD (*n* = 5) | | | |
